# Imipramine: Do Metabolite Plasma Levels Affect Treatment Response?

**DOI:** 10.1097/JCP.0000000000002187

**Published:** 2026-04-27

**Authors:** Roan Y.N. Kasanmonadi, Astrid M. Kamperman, Birgit C.P. Koch, Tom K. Birkenhager

**Affiliations:** 1 Department of Psychiatry, Erasmus Medical Center; 2 Department of Hospital Pharmacy, Erasmus Medical Center, Rotterdam, The Netherlands

**Keywords:** imipramine, desipramine, therapeutic drug monitoring, depression, pharmacokinetics

## Abstract

**Purpose/Background:**

Although combined plasma levels of imipramine and its metabolite desipramine are routinely assessed during therapeutic drug monitoring (TDM) of imipramine treatment, individual plasma levels are often overlooked. Imipramine and desipramine differ in their pharmacokinetic properties. Previous research in patients with generalized anxiety disorder suggested that imipramine treatment efficacy might be negatively influenced by high desipramine levels. We thus explored the relationship between imipramine and desipramine levels and treatment response in depressed inpatients.

**Methods/Procedures:**

This exploratory retrospective study involved 70 inpatients with major depression disorder who had undergone 5-week imipramine treatment. Weekly combined levels were measured, and adjustments were made until adequate levels were reached. We assessed response rates on the 17-item Hamilton Rating Scale for Depression (HDRS_17_) and used Mann-Whitney *U* tests to compare imipramine and desipramine plasma levels between responders and nonresponders.

**Findings/Results:**

After 5 weeks of treatment, nonresponders had significantly higher desipramine levels (*P* = 0.015) and combined plasma levels (*P* = 0.020) than responders. Imipramine levels did not differ significantly between groups. Plasma levels did not differ significantly between patients with and without concomitant CYP2D6- or CYP2C19-inhibiting medication.

**Implications/Conclusions:**

By showing that desipramine plasma levels were higher in nonresponders to imipramine treatment, our findings imply that desipramine negatively impacts imipramine’s therapeutic effect. When performing TDM, it may be important to consider metabolite plasma levels as well as combined plasma levels.

Treatment of major depression often involves several antidepressant drugs and several classes of these drugs. Tricyclic antidepressants (TCA), such as imipramine, are useful for treating severe depressive disorders.^1^ As treatment with imipramine is intended to achieve therapeutic drug levels, clinicians can use therapeutic drug monitoring (TDM) to adapt doses (as with other TCAs) according to the plasma levels they find. While the risk of adverse effects increases if the levels are too high, as demonstrated by Preskorn and colleagues, the efficacy of antidepressants decreases if they are too low.^2,3^ Since the metabolism of TCAs varies greatly between patients, TDM plays a very important part in treatment with imipramine,^4^ as it helps to maximize efficacy and prevent adverse effects, which are known to increase the odds that a patient will discontinue medication. While antidepressants typically require 4 to 6 weeks to demonstrate their efficacy, earlier responses have also been documented.^5–7^

Imipramine is mainly metabolized by CYP2C19 into its active metabolite, desipramine, which is metabolized by CYP2D6 into the inactive 2-hydroxy-desipramine.^8,9^ See Figure 1 for a schematic illustration of the metabolic pathways of imipramine.^10^ Desipramine itself is not only a metabolite of imipramine, but it has also proved to be an effective antidepressant whose pharmacokinetic properties are comparable with those of nortriptyline.^10,11^

**FIGURE 1:**
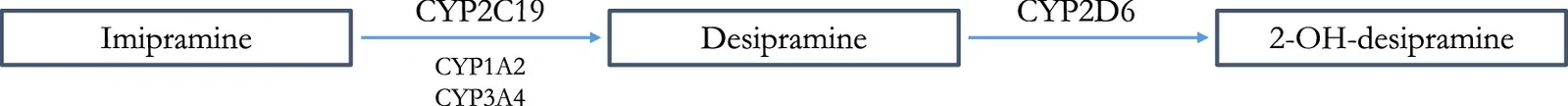
Schematic overview of the metabolic pathway of imipramine.

In TDM, the active moiety, which is the sum of imipramine and desipramine plasma levels, is assessed to make dose adjustments. A recommended reference range of 175 to 300 µg/L has been proposed for these combined plasma levels, its upper limit being based on adverse effects rather than efficacy.^2^ However, since imipramine has a relatively strong affinity for the 5-hydroxytryptamine (5-HT) receptor and desipramine has a relatively strong affinity for the noradrenaline (NA) receptor, it is not obvious that their antidepressant efficacy is similar.

This begs the question of whether either the separate metabolite plasma levels or the ratio between them is relevant in achieving optimal efficacy. While McLeod et al^12^ reported in a sample with patients with generalized anxiety disorder that desipramine plasma levels correlated negatively with clinical efficacy, they found no correlation between imipramine plasma levels and clinical efficacy. To the best of our knowledge, such relationships have not yet been described in samples with depressed patients.

## AIM OF THE STUDY

This study aimed to explore the relationship between separate imipramine and desipramine levels and treatment response by comparing imipramine and desipramine levels between responders and nonresponders after 5 weeks of treatment with imipramine.

## METHODS

### Subjects

The Depression Unit at Erasmus MC is specialized in the treatment of severely depressed and refractory depressed patients. Upon admission, these patients undergo a routine drug-free observation period of at least 1 week, followed by assessment of symptom severity using the 17-item Hamilton Rating Scale for Depression (HDRS_17_).^13^ The possible start of treatment with antidepressants, such as imipramine, is then discussed with the patient and a relative. To ensure medication adherence, imipramine is administered by and taken under supervision of the nursing staff. The clinicians at the ward are trained in imipramine management. Depression severity is routinely assessed using the HDRS_17_. Imipramine plasma levels are measured weekly, and doses are adjusted based on measured combined levels until therapeutic combined levels (200 to 300 µg/l, according to the local treatment protocol) are achieved. To take the half-life of imipramine into account and ensure that steady-state levels are assessed, venipuncture takes place 5 days after starting imipramine or dose adjustments, and 12 hours after ingestion of imipramine. Blood samples are sent to the laboratory on the day of venipuncture. Analysis and report of plasma levels takes place the next day. If necessary, doses are adjusted based on attaining therapeutic combined plasma levels on the day of the report. After therapeutic plasma levels have been attained, doses do not generally need to be adjusted.

The patients included in this study were admitted to the Depression Unit, diagnosed with major depression, and treated with imipramine over the period between January 1, 2010 and October 3, 2021. Inclusion criteria were age 18 years or older; monotherapy with imipramine after a drug-free observation period; adequate treatment with imipramine over a period of 5 weeks, as shown by therapeutic imipramine plasma levels; and severe or very severe major depression operationalized as an HDRS_17_ score of >19 at baseline. Exclusion criteria were missing data (fewer than 21 days of follow-up after attainment of therapeutic plasma levels; the lack of imipramine plasma levels that would indicate adequate dosage; or missing HDRS_17_ data).

Follow-up was set at 35 days after the first administration of an adequate dose. We accepted a follow-up between 21 and 56 days, since early response to antidepressants has been documented as well.^7,14,15^

For patients admitted and treated with imipramine several times within the specified timeframe, only the first treatment period was included.

### Variables

To build our database, we combined the available imipramine levels measured at Erasmus MC with information extracted from patient charts. Response was defined as ≥ 50% reduction in the HDRS_17_. Due to the relatively short follow-up, we opted not to categorize by remission, as patients in our Depression Unit are often discharged after achieving response but before achieving full remission. Reduction in the HDRS_17_ was calculated by subtracting baseline scores from end-of-treatment scores. Baseline scores were derived from the last available score measured on the HDRS_17_ before treatment initiation. End-of-treatment scores were as close to 35 days as possible.

Our variables of interest were separate imipramine and desipramine plasma levels, combined plasma levels, and the metabolic ratio (MR), which was defined as the ratio between desipramine plasma levels and imipramine plasma levels. These variables are also presented in Figure 2.

**FIGURE 2:**
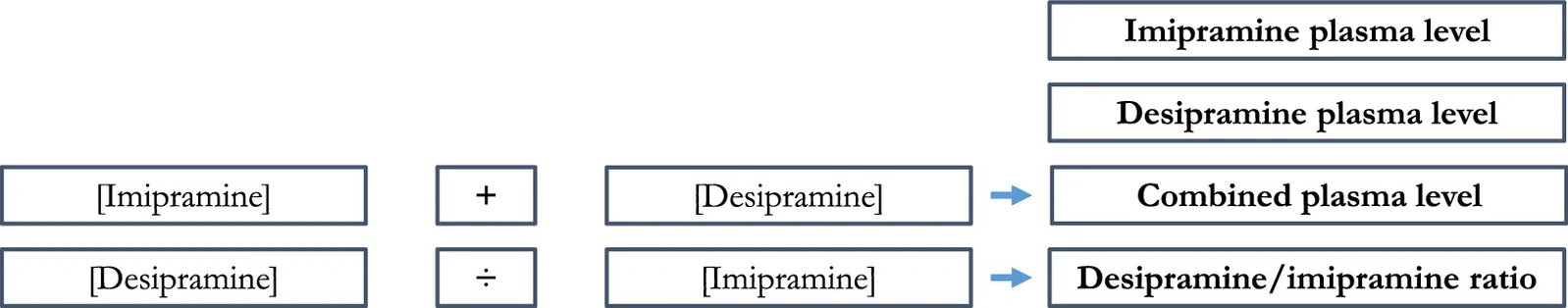
Schematic explanation of the outcome variables assessed in our analysis.

Plasma levels are evaluated repeatedly at our Depression Unit. In our analysis, we used plasma levels that had been measured when combined plasma target levels were achieved. Assays were conducted using a validated ultra-pressure liquid chromatography-tandem mass spectrometry (UPLC-MS/MS) method.^16^ Adequate dose is defined as the dose that corresponds to the target plasma levels of imipramine and desipramine combined. According to the treatment protocol, combined plasma levels in this study were set at >200 µg/L.

As adverse effects had not been recorded routinely or systematically, we did not evaluate them.

### Ethics

The use of data was permitted by the Medical Ethics Review Committee of the Erasmus MC, University Medical Center, under non-WMO (Medical Research Involving Human Subjects Act) regulations. Due to the retrospective nature of this study, written informed consent was not obtained.

### Statistical analysis

For our analysis, we explored the relationship between imipramine levels, desipramine levels, and response by comparing imipramine and desipramine levels between responders and nonresponders to imipramine treatment. For our statistical analysis, we used Statistical Package for Social Science version 25.0. Normality was assessed visually using histograms, after which the means of numerical data were compared using an unpaired *t* test, or, in cases of non-normal distribution, a Mann-Whitney *U* test. Depending on the cell count, our comparisons of categorical data used either χ^2^ tests or Fisher exact test. A *P*-value of <0.05 was considered significant.

## RESULTS

### Patient Sample

Although the imipramine plasma levels of 264 patients were available, 119 patients did not meet the inclusion criteria. Eighty-five were excluded due to missing data or <21 days of follow-up. Ten others were excluded due to the lack of a drug-free observation period after admission (n = 4), concomitant lithium use (n = 4), and second admission after successful previous treatment with imipramine at the Depression Unit at Erasmus MC (n = 2).

Eventually, 70 patients were analyzed. Figure 3 presents a flowchart detailing the inclusion and exclusion process.

**FIGURE 3:**
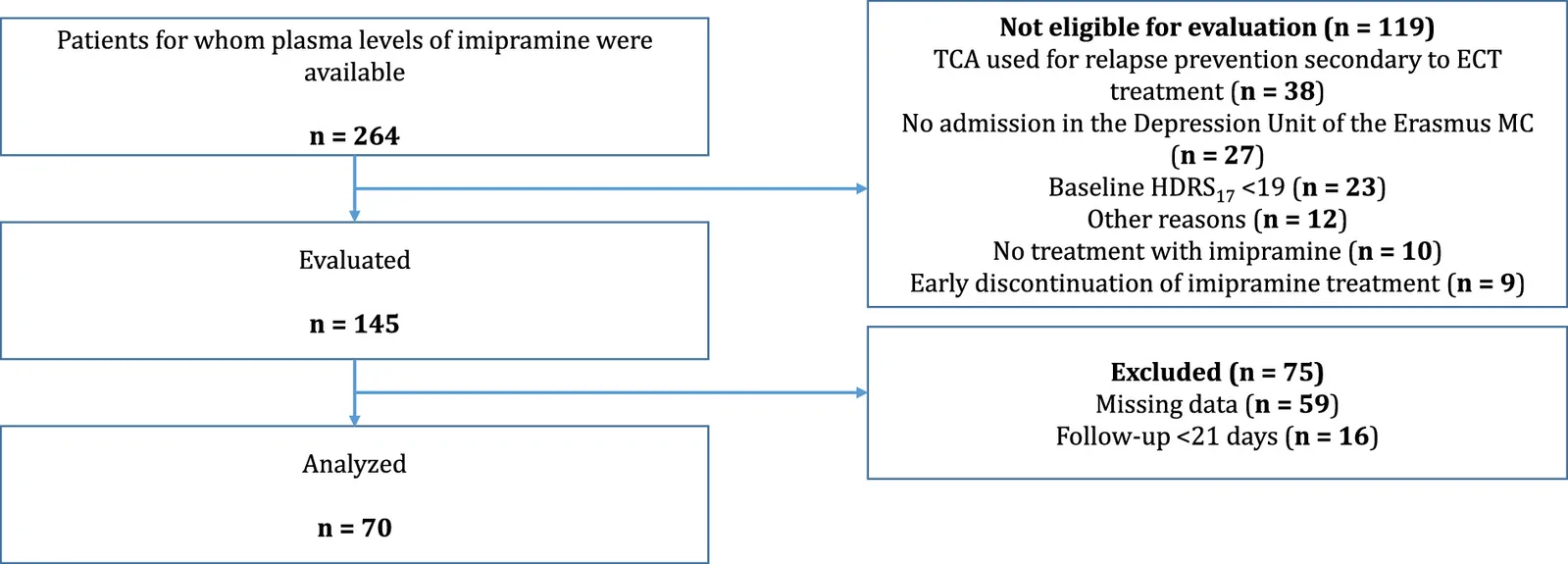
Overview of the inclusion and exclusion process.

### Baseline Characteristics

The mean age of our sample was 55.7 years (SD = 8.5), which was consistent with the average age of depressed patients in our Depression Unit. Slightly over half of our sample (57.1%) consisted of female patients. The daily doses administered ranged from 75 to 400 mg. The mean dose in our response group was 224.2 milligrams; in our nonresponse group, it was 191.5 milligrams. Average follow-up was 35.4 days. Twenty-4 patients used concomitant CYP2D6 or CYP2C19-influencing medication. Table 1 shows a complete overview of the baseline characteristics for this sample.

**TABLE 1:** Baseline Characteristics

	All (n = 70)	Nonresponse (n=38)	Response (n=32)	*P*
Age ± SD, y	55.7 ± 8.5	55.5 ± 9.4	55.9 ± 7.5	0.660
Sex, n (%)				0.269
Male	30 (42.9)	16 (42.1)	14 (43.8)	
Female	40 (57.1)	22 (57.9)	18 (56.2)	
Dose ± SD, mg	206.4 ± 74.9	191.5 ± 70.3	224.2 ± 77.3	0.042
Baseline HDRS17 score ± SD	25.4 ± 4.2	25.0 ± 3.8	25.8 ± 4.6	0.157
Final HDRS17 score ± SD	14.9 ± 8.5	21.4 ± 5.1	7.1 ± 3.8	<0.001
Duration of follow-up ± SD, days	35.4 ± 7.0	34.6 ± 6.4	36.4 ± 7.7	0.515
Concomitant CYP-influencing medication^*^ (%)	24 (34.3)	16 (42.1)	8 (25)	0.133

Data are presented as mean values.

^*^ CYP2D6- or CYP2C19-influencing medication.

In total, 24 patients used CYP2D6- or CYP2C19-inhibiting medication, 3 of whom used both. Six patients used CYP2D6-inhibiting medication (3 using haloperidol, 2 using metoclopramide, and 1 using methadone due to neuropathic pain). Twenty-one patients used CYP2C19-inhibiting medication (omeprazole, esomeprazole, pantoprazole, ethinylestradiol/levonorgestrel, cyprotion/ethinylestradiol). All these inhibitors were either weak inhibitors or in vitro only inhibitors.^17^

### Outcome Variables

Desipramine levels were significantly higher in nonresponders than in responders. Combined plasma levels also differed significantly, with a median value of 278 µg/l across both groups, 289.5 µg/l in the nonresponse group, and 257 µg/L in the response group (*P* = 0.020). Imipramine levels and desipramine/imipramine ratios did not differ significantly between groups. Table 2 presents an overview of our outcome variables.

**TABLE 2:** Pharmacokinetic Differences Between nonresponders and Responders

	All (n = 70)	Nonresponse (n = 38)	Response (n = 32)	*P*
First adequate plasma level, µg/L				
Imipramine	142.5 (120.8–172.8)	138 (122.8–172.8)	152.5 (109.5–177.3)	0.377
Desipramine	135.5 (91.5–183.5)	152.5 (109.8–196.5)	113.5 (80.3–160.8)	0.015
Combined plasma levels	278 (243.0–316.3)	289.5 (261.5–316.3)	257 (226.3–315.0)	0.020
Desipramine/imipramine ratio	0.97 (0.6–1.5)	1.10 (0.6–1.16)	0.80 (0.5–1.3)	0.075

Data are presented as median values with interquartile ranges.

It is known that most psychiatric drugs are metabolized by several cytochrome P450 (CYP) enzymes.^2^ CYP enzyme activity can also be influenced by certain medications, leading to either inhibition or induction.^17^ In our population, several patients also used medications other than imipramine, some of which influenced CYP2D6 or CYP2C19 activity. This meant that concomitant medication might influence imipramine levels in these patients. To take into account possible CYP2D6 or CYP2C19-influencing medication, we, therefore, established which patients had used such medication, divided them into groups on this basis, and then compared these groups in terms of changes on the HDRS_17_. After performing Mann-Whitney *U* tests, we found no significant differences in combined plasma levels (*P* = 0.958), imipramine plasma levels (*P* = 0.914), desipramine plasma levels (*P* = 0.470), or desipramine/imipramine ratios (*P* = 0.519) between patients who did and did not use concomitant CYP2D6- or CYP2C19-inhibiting medication.

There was no significant difference in the proportion of nonresponders between patients with plasma levels of 200 to 300 µg/L and those with levels >300 µg/L [χ^2^(1) = 0.22, *P* = 0.64].

## DISCUSSION

Between responders and nonresponders to imipramine treatment, our study found notable differences in the plasma levels of desipramine and combined imipramine and desipramine. Specifically, desipramine levels (152.5 vs 113.5 µg/l) and combined levels (289.5 vs 257 µg/l) were significantly higher in nonresponders. Imipramine levels between groups did not differ significantly between groups.

These findings suggest that higher desipramine levels have a negative impact on the efficacy of imipramine. Such an impact was also suggested, albeit not at a level of statistical significance, by a median metabolic ratio of <1 in responders and >1 in nonresponders.

One might expect an overrepresentation of nonresponders among subjects with higher plasma concentrations, as flexible dosing regimens often result in higher dosing for patients who do not respond to treatment.^18^ However, in our sample, we found no significant difference in response rates between patients with plasma levels of 200 to 300 µg/l and those with levels >300 µg/l. Moreover, baseline characteristics showed that mean doses were actually higher in the response group. This suggests that, at least in this cohort, higher combined imipramine plasma levels were not driven by treatment resistance.

Thirty-two of the 70 patients in our sample (45.7%) were responders to imipramine, and 18 (25.7%) were remitters. This is consistent with the response rates of 39.5% to 51.4% and remission rates of 23% found in past studies of depressed inpatients at Erasmus MC.^19–21^

### Comparison With Previous Reports

Our findings are consistent with earlier research by McLeod et al,^12^ who found a similar relationship between desipramine levels and treatment efficacy in generalized anxiety disorder. These authors suggested that, possibly due to the influence of the different receptor mechanisms, the therapeutic effects of imipramine were counteracted by the anticholinergic activity of desipramine. The prevalence of preexisting anxiety levels could be relevant for the treatment response in our sample. However, we did not possess data on anxiety levels or generalized anxiety disorders. Whereas imipramine is a combined serotonin and noradrenaline reuptake inhibitor, desipramine is mainly a noradrenaline reuptake inhibitor.^10^ However, since desipramine is also an effective antidepressant on its own, we cannot simply state that it counteracts imipramine efficacy through its noradrenergic or anticholinergic effects.^11,22–24^ This begs the question of why it would counteract the efficacy of imipramine treatment. One possible explanation is the existence of a therapeutic window for desipramine plasma levels. To date, only a linear relationship between desipramine levels and the efficacy of treatment with desipramine has been described, but a therapeutic window has not been found.^25^

In clinical practice, a therapeutic window is used in treatment with nortriptyline, whose pharmacokinetic properties are similar to those of desipramine. Lower limits between 49 and 58 µg/l and upper limits between 139 and 170 µg/l have been reported.^24,26–29^ Since desipramine and nortriptyline are pharmacokinetically alike, one might suppose that such a window could also exist for desipramine. Two studies have described a lower limit of 75 µg/l of desipramine levels in imipramine treatment.^24,30^ In our sample, 6 patients, 5 of whom were responders, had desipramine levels lower than 75 µg/l.

In the treatment of refractory obsessive-compulsive disorders with clomipramine, the CYP2C19 inhibitor fluvoxamine can be administered to inhibit methylation of clomipramine and secure high clomipramine plasma levels, but low desmethylclomipramine levels. If our findings are replicated in a larger, prospective study, this augmentation could be a possibility for nonresponders to imipramine treatment in inpatients with major depressive disorder.^31,32^

### Strengths and Limitations

A major strength of this study is the population selected. Our analysis was performed in a homogeneous group of depressed inpatients in a controlled setting where medication adherence was optimized, patients were treated according to standard treatment protocols, and efficacy was properly assessed. The homogeneity of our group was ensured by the drug-free observation period, after which imipramine treatment would start only for patients with clinical and severe major depression. When the severity of depression symptoms and the efficacy of treatment are evaluated, use of the HDRS_17_ ensures consistency and reliability.

Apart from the study performed by McLeod et al,^12^ no other study on the relationship between separate imipramine and desipramine levels and the efficacy of imipramine treatment has been performed, even though, in clinical practice, it is important to optimize TDM in treatment with antidepressants. Ours is the first study to assess the role of these plasma levels in depressed inpatients.

One limitation is our retrospective study design, which was a consequence of our dependence on the completeness of the patient charts. Due to missing data—which often meant that no baseline or end-of-treatment HDRS_17_ score was reported—58 patients were excluded, substantially affecting the size of our sample.

A second limitation is the flexible follow-up period (21 to 56 d) that preceded assessment of imipramine efficacy. Clinical efficacy is generally assessed after 4 to 6 weeks of imipramine treatment. Even though one might think that this could lead to underrepresentation of responders, some of whom might otherwise benefit from a longer treatment period, early response to antidepressants has also been documented.^7,14,15^

Finally, Glassman et al^33^ reported that depressed patients with delusions may display less response than depressed patients without delusions. Unfortunately, we did not have specific diagnostic information regarding psychotic features. Menopausal status may also influence imipramine response, as described by Kornstein et al.^34^ However, in our sample, of the 40 women included, 28 were older than 50, with a minimum age of 37 years. Therefore, our sample is likely composed predominantly of postmenopausal women.

## CONCLUSION

In this explorative retrospective study, our finding of significantly higher desipramine plasma levels in nonresponders to imipramine treatment than in responders underscores the potentially negative impact of higher desipramine plasma levels on the therapeutic efficacy of imipramine in depressed inpatients.

### Future Prospects

Given large sample sizes, prospective studies with fixed follow-up periods that assess the influence of specific desipramine and imipramine plasma levels on clinical response might help to estimate the effect size of our reported findings. If these relationships were clarified, better-tailored, more effective approaches to treating depressive disorders might result
